# Clinical outcomes and psychosomatic correlates of integrative inpatient care based on Traditional Korean Medicine for acute neck and low back pain following traffic accidents: a retrospective cohort study

**DOI:** 10.3389/fmed.2025.1684034

**Published:** 2025-11-13

**Authors:** Yohwan Kim, Yejin Hong, Suji Lee, Tae-Hun Kim, Dongwoo Nam, Seunghoon Lee

**Affiliations:** 1 Department of Clinical Korean Medicine, Graduate School, Kyung Hee University, Seoul, Republic of Korea; 2 Department of Acupuncture and Moxibustion, Kyung Hee University College of Korean Medicine, Kyung Hee University Medical Center, Seoul, Republic of Korea; 3 Korean Medicine Clinical Trial Center, Korean Medicine Hospital, Kyung Hee University, Seoul, Republic of Korea

**Keywords:** traffic accident, whiplash-associated disorders, Traditional Korean Medicine, psychosomatic factors, retrospective cohort study

## Abstract

**Introduction:**

Traffic accidents (TAs) frequently cause acute neck and low back pain accompanied by psychosomatic symptoms. This study evaluated the clinical utility and safety of Traditional Korean Medicine as part of integrative care for acute axial pain following TAs and explored how psychosomatic factors influence recovery.

**Methods:**

Inpatient cohort data from patients with TA-related neck or low back pain, collected between September 2018 and March 2023, were analyzed. Patients received semi-standardized Traditional Korean Medicine treatments, including acupuncture, pharmacopuncture, cupping, herbal medicine, and Chuna manual therapy. Axial pain was defined as a higher Numeric Rating Scale score for either neck or low back pain. We assessed changes in pain, function, psychosomatic symptoms, and quality of life and analyzed the associations between pain and psychosomatic factors.

**Results:**

One hundred ninety patients were analyzed. Significant improvements were observed, with mean changes in axial, neck, and low back pain of 2.09, 2.21, and 1.94 (all *p* < 0.05). On admission, the axial pain Numeric Rating Scale scores were positively correlated with the Insomnia Severity Index and Patient Health Questionnaire-9. Greater reductions in axial pain, as measured by Numeric Rating Scale scores, were significantly associated with decreases in scores on the Beck Anxiety Inventory, Fatigue Severity Scale, Patient Health Questionnaire-9, and Insomnia Severity Index in regression analyses adjusted for clinical and demographic covariates. No serious adverse events were reported, and blood test results were normal.

**Conclusion:**

Traditional Korean Medicine appears to be an effective and safe treatment for acute neck and low back pain after TAs. Moreover, improvements in psychosomatic symptoms may have contributed to the improved pain-related outcomes.

## 1 Introduction

The acceleration–deceleration mechanism commonly involved in traffic accidents (TAs) can lead to various musculoskeletal injuries, including whiplash-associated disorders (1), which frequently manifest as neck and low back pain (2). These conditions can significantly affect the patients’ quality of life and functional status (3) and often require comprehensive treatment during the acute recovery phase (4, 5). Addressing musculoskeletal pain early after a motor vehicle accident is crucial to prevent the development of chronic pain. Approximately half of the individuals who sustained whiplash injury fully recovered, whereas the other half continued to experience persistent neck-related disability or psychological impairment (6, 7). Recovery did not follow a linear trajectory; instead, it tended to plateau, with minimal improvement occurring after the initial phase (8).

Conventional pharmacological therapies, such as nonsteroidal anti-inflammatory drugs, non-opioid analgesics, and opioids, may be prescribed for acute pain resulting from TAs. However, the most effective intervention for preventing progression to chronic pain remains unclear (9). In contrast, non-pharmacological therapies have shown promise in managing whiplash-associated disorders (10). Traditional Korean Medicine (TKM), a form of complementary and alternative medicine, has been widely used in Korea as an adjunct to conventional treatment for managing musculoskeletal pain following TAs (11, 12). Several studies have reported the potential benefits of TKM interventions for TA-related musculoskeletal disorders, including pain reduction (13), functional improvement (14), and enhanced patient satisfaction (15). However, most of the existing literature focuses on targeting specific symptoms or evaluating the efficacy of a single intervention, thereby limiting its applicability in real-world clinical practice. In clinical settings, patients with TA-related injuries frequently present with pain in multiple regions, most commonly the neck and low back, rather than with isolated symptoms. According to a population-based cohort study, only 0.4% of the patients with TA report pain confined to the posterior neck area (2). Moreover, low back pain is as prevalent as neck pain, and nearly one in four patients experience both simultaneously (16). Furthermore, most patients receive combined TKM treatments rather than a single intervention (17). This discrepancy between research conditions and clinical practice highlights the need for studies that reflect the complexity of real-world treatment environments.

Additionally, growing evidence suggests that psychosomatic factors influence both pain perception and treatment response (18). In clinical practice, depression and anxiety are known to markedly amplify patients’ pain (19). Among patients with TA-related injuries, early psychological factors, such as post-traumatic stress, may adversely affect prognosis, potentially contributing to prolonged symptoms and delayed recovery (20). Existing evidence suggests that the interaction between physical injury and psychological factors may contribute to the development of chronic whiplash-associated disorder (21, 22). Therefore, understanding pain outcomes requires a comprehensive perspective that considers not only physical symptoms, but also psychological and somatic factors that may influence the perception and management of pain. However, few studies have systematically evaluated the role of psychosomatic factors in the context of integrative care, which combines pharmacological therapy and TKM for TA-related injuries.

Given these considerations, it is essential to investigate how psychosomatic variables interact with therapeutic outcomes in clinical practice. To this end, this study retrospectively analyzed real-world data from patients hospitalized for neck or low back pain after TA to evaluate the clinical utility and safety of integrative interventions during acute recovery. Furthermore, adopting a biopsychosocial framework, we investigated how changes in psychosomatic symptoms such as depression, anxiety, insomnia, and fatigue are associated with improvements in pain perception and recovery.

## 2 Materials and methods

### 2.1 Clinical data registry

The clinical data of patients hospitalized and treated at the Department of Acupuncture and Moxibustion at Kyung Hee University Korean Medicine Hospital were systematically recorded in the Kyung Hee ACUpuncture REGistry (KH-ACUREG). The KH-ACUREG systematically collects clinical data from both inpatients and outpatients who receive treatment at the Spine and Joint Center, Department of Acupuncture and Moxibustion, Kyung Hee University, Korean Medicine Hospital. The registry includes key data, such as diagnoses, chief complaints, pain and functional assessments, and records of any adverse events following treatment. This secure registry is maintained within a restricted-access document repository, accessible exclusively to authorized medical personnel in the department, thereby ensuring the confidentiality and integrity of all clinical information.

The study protocol was approved by the Institutional Review Board (IRB) of Kyung Hee University Korean Medicine Hospital (KOMCIRB 2021-09-015-005). As this study was a retrospective analysis using previously collected data and documents that posed no direct risk or disadvantage to participants, the requirement for written informed consent was waived by the IRB.

### 2.2 Selection of patients

In this study, we identified patients with TA-related injuries registered in the KH-ACUREG. A list of these patients was submitted to the medical record management team of the hospital, which retrieved the corresponding clinical data. Patients who met all the inclusion criteria were selected to participate in the study. Relevant clinical information was extracted from three sources: KH-ACUREG, Electronic Medical Records (EMR), and paper-based medical charts.

To be eligible for inclusion, the patients had to be at least 18 years old and have received inpatient treatment for a period ranging from 2 to 28 days. In addition, only patients admitted within 21 days of the onset of TA were included. Eligible participants were diagnosed with whiplash-associated disorder grades 1–3, without any fractures, and reported a baseline pain intensity of 4 or higher on the Numeric Rating Scale (NRS) as their chief complaint.

Patients were excluded if they did not receive TKM treatment during their hospital stay, had insufficient outcome measures to compare pre- and post-treatment statuses, or had a history of surgery or trauma affecting the area of interest. Patients with significant comorbidities that could potentially influence treatment outcomes—such as inflammatory arthritis (e.g., rheumatoid arthritis, ankylosing spondylitis, or psoriatic arthritis), fibromyalgia, severe neurological impairment, or major psychiatric disorders unrelated to the accident—were excluded. Furthermore, for patients who were hospitalized more than once during the study period, only data from their first admission were included in the analysis, and subsequent admissions were excluded. Finally, patients deemed unsuitable for retrospective medical record analysis at the discretion of the researchers were excluded. As this was a retrospective study using registry data, no formal sample size calculation was performed. All patients registered in the KH-ACUREG during the study period who met the inclusion and exclusion criteria were included in the analysis.

### 2.3 Integrative inpatient care

Patients hospitalized for musculoskeletal pain following TA in the Department of Acupuncture and Moxibustion at Kyung Hee University Korean Medicine Hospital received semi-standardized integrative care combined with pharmacological therapy and TKM treatments. TKM treatments typically consist of acupuncture, electroacupuncture, pharmacopuncture, cupping therapy, herbal medicine, and Chuna manual therapy. Each treatment regimen was individualized according to the symptoms and clinical presentation of the patient. While TKM treatments were prioritized, pharmacological therapy such as analgesics was provided at the minimum effective dose when additional pain control was required.

#### 2.3.1 Acupuncture and electroacupuncture

Acupuncture was performed twice daily using disposable stainless-steel needles. The primary needle size was 0.25 × 40 mm (Dongbang Medical Co., Ltd., Seongnam, Republic of Korea), while 0.20 × 30 mm or 0.40 × 60 mm needles were used based on the anatomical location. For each pain site, both Ashi points (阿是穴) and specific acupoints were targeted. In cases of neck pain, commonly used acupoints include Yamen (GV15, 啞門), Tianzhu (BL10, 天柱), and Jianjing (GB21, 肩井). For low back pain, acupoints such as Shenshu (BL23, 腎兪), Yaoyangguan (GV3, 腰陽關), Dachangshu (BL25, 大腸兪), and Mingmen (GV4, 命門) were utilized. When psychosomatic symptoms such as insomnia or anxiety were present, additional acupoints were selected, including Neiguan (PC6, 內關), Zusanli (ST36, 足三里), and Hegu (LI4, 合谷). Needles were inserted to a depth of 10–20 mm and retained for 20 min during each session. During the second acupuncture session each day, electroacupuncture was performed using an STN-111 device (StraTek Co., Ltd., Anyang, Republic of Korea). Two channels were applied to each pain site using the same acupoints, as previously described. Stimulation was delivered at 2 Hz for local points and at 100 Hz for distal points. The intensity of stimulation was adjusted to the highest level tolerable by the patient without causing discomfort.

#### 2.3.2 Pharmacopuncture

Depending on the symptoms of the patient, either bee venom or Hominis placental pharmacopuncture was performed. Before bee venom pharmacopuncture, a skin test was conducted to confirm a negative allergic reaction. A 0.5 mL dose of bee venom, diluted at a ratio of 1:30,000, was injected into five to six acupuncture points in the affected area using a disposable insulin syringe (30 gauge, Hwajin Medical Co., Seoul, Republic of Korea). The solution was prepared by dissolving 10 mg of dried bee venom (Yoomil Garden, Hwasun, Republic of Korea) in 300 mL of saline (Joongwe Pharmaceuticals, Seoul, Republic of Korea). Similarly, a single vial (2 mL) of Hominis placental extract (5 mg per vial, Girin External Herbal Dispensary, Wonju, Republic of Korea) was administered at five to six acupuncture points in the affected area using the same type of syringe.

#### 2.3.3 Cupping therapy

Cupping therapy, including both dry and wet techniques, was administered on alternating days. For patients with cervical pain, treatment was administered at acupoints in the cervical region, whereas those with lumbar pain received treatment at acupoints in the lumbar region. Cupping therapy was administered to both regions in patients with cervical and lumbar pain. The wet cupping therapy involved puncturing the targeted acupoints with a lancet, followed by the application of negative pressure using disposable sterile cups (Dongbang Medical Co., Ltd., Seongnam, Republic of Korea) for 5 min.

#### 2.3.4 Herbal medicine

Based on the symptoms of the patient, three types of herbal medicines were prescribed to alleviate pain, promote blood circulation, and reduce blood stasis. For patients with severe lumbar pain, *Tonghyeol hwallak-tang* (統血活絡湯) was commonly prescribed, while *Tonghyeolhoesu-san* (統血回首散) was used for significant cervical pain. In cases of extreme fatigue or weakness, *Tonghyeolssanghwa-tang* (統血雙和湯) was administered. Herbal prescriptions were tailored to the individual condition of the patient and reported symptoms. Patients were instructed to consume one pack of herbal medicines three times daily. Each pack contained 100 mL of the decoction (Supplementary Table S1).

#### 2.3.5 Chuna manual therapy

Chuna manual therapy was performed by licensed TKM doctors with a minimum of 2 years of clinical experience. This therapy involves hands-on techniques, including mobilization, traction, and manipulation, specifically targeting the cervical and lumbar spines. In addition, specialized approaches such as the Muscle Energy Technique, Strain-Counterstrain Technique, and Soft Tissue Manipulation have been applied to areas affected by pain. Therapy sessions were conducted daily, with each session lasting approximately 15–30 min, depending on the condition of the patient and their response to treatment.

### 2.4 Outcomes

To evaluate the treatment effectiveness, several patient-reported outcomes and clinical parameters were assessed at both admission and discharge. These included the measurement of pain intensity, functional disability, quality of life, psychosomatic symptoms, and routine blood tests.

#### 2.4.1 Pain intensity

Pain intensity was measured using the NRS, a single-item, 11-point scale, ranging from 0 (“no pain”) to 10 (“worst imaginable pain”). Patients were asked to rate their current level of pain based on a numerical continuum (23).

#### 2.4.2 Neck disability

The Neck Disability Index (NDI) is a 10-item questionnaire designed to assess the impact of neck pain on daily activities, including personal care, work, and concentration. Each item is scored on a scale of 0–5, resulting in a total score ranging from 0 to 50, with higher scores indicating greater functional disability (24). The Korean version of the NDI was used in this study (25).

#### 2.4.3 Low back disability

The Oswestry Disability Index (ODI) is a 10-item questionnaire that assesses the level of disability associated with low back pain, covering various domains such as pain intensity, walking, sleeping, and daily function. Each item is rated on a scale of 0–5, and the total score is converted into a percentage ranging from 0 to 100%, with higher percentages indicating more severe disability (26). The Korean version of the ODI was used in this study (27).

#### 2.4.4 Insomnia

The Insomnia Severity Index (ISI) is a 7-item questionnaire that assesses the severity of insomnia and its impact on daily functioning. Each item is rated on a scale of 0–4, yielding a total score ranging from 0 to 28, with higher scores indicating more severe insomnia. A score of 0–7 indicates no clinically significant insomnia, whereas scores between 22 and 28 indicate severe insomnia (28). The Korean version of the ISI (ISI-K) was used in this study (29).

#### 2.4.5 Anxiety

The Beck Anxiety Inventory (BAI) is a 21-item self-report questionnaire designed to measure the severity of anxiety symptoms, particularly somatic manifestations such as trembling, palpitations, and shortness of breath. Each item is rated on a scale of 0–3, resulting in a total score ranging from 0 to 63, with higher scores indicating greater severity of anxiety. Scores between 0 and 7 indicate minimal anxiety, whereas scores between 30 and 63 indicate severe anxiety (30). The Korean version of the BAI was used in this study (31).

#### 2.4.6 Depression

Depressive symptoms were assessed using the Patient Health Questionnaire-9 (PHQ-9), a 9-item instrument based on The Diagnostic and Statistical Manual of Mental Disorders-5 criteria. Each item is rated on a scale from 0 to 3, yielding a total score ranging from 0 to 27, with higher scores indicating greater severity of depression. A score of 0–4 indicates minimal depression, whereas a score of 20–27 indicates severe depression (32). The Korean version of the PHQ-9 was used in this study (33).

#### 2.4.7 Fatigue

The Fatigue Severity Scale (FSS) is a 9-item questionnaire designed to assess the impact of fatigue on daily functioning. Each item was rated on a 7-point Likert scale, with a total score ranging from 9 to 63. Higher scores indicate more severe fatigue (34). The Korean version of the FSS was used in this study (35).

#### 2.4.8 Quality of life

Quality of life was measured using the EuroQol five-dimensional questionnaire (EQ-5D, three-level version), which is a standardized instrument that measures health-related quality of life across five dimensions: mobility, self-care, usual activities, pain/discomfort, and anxiety/depression. Each dimension has three levels of severity represented numerically as follows: 1 = no problems, 2 = some problems, and 3 = extreme problems. The Korean version of the EQ-5D was used in this study (36). The total score was calculated by summing the scores across the five dimensions.

#### 2.4.9 Adverse events

Liver and kidney functions, including aspartate aminotransferase (AST), alanine aminotransferase (ALT), alkaline phosphatase (ALP), total bilirubin, blood urea nitrogen (BUN), and creatinine levels, were evaluated using blood tests. Procedure-related adverse events such as erythema, bleeding, and swelling at the treatment sites were closely monitored throughout the hospitalization period. When such events occurred, they were documented in both the EMR and paper-based charts.

### 2.5 Statistical analysis

Axial pain was defined as a higher value on the NRS scale between the neck and low back pain scores when both were available. If only one of the two scores was recorded, the score was used to represent axial pain.

Descriptive statistics were used to summarize the baseline characteristics, including means, standard deviations, frequencies, and percentages. Pre- and post-treatment differences were assessed for statistical significance using a paired *t*-test. Correlations between baseline pain intensity and psychosomatic variables were analyzed using Pearson’s correlation analysis. Simple linear regression analyses were conducted to assess the influence of psychosomatic and other clinical factors on baseline NRS scores. Additionally, to evaluate the treatment outcomes, both simple and multiple linear regression analyses were performed using the NRS scores at discharge as the dependent variable. To systematically assess the independent contribution of psychosomatic factors, we employed a hierarchical multiple linear regression with three prespecified models. This approach allows us to observe how the association changes as we control for different sets of variables. Model 1 was adjusted for the initial NRS pain score only to establish a baseline association. Model 2 was additionally adjusted for key demographic and clinical characteristics; these covariates were selected based on prior literature suggesting their potential influence on pain outcomes after traffic accidents. Finally, Model 3 included hospitalization duration to isolate the effect of psychosomatic improvement from the influence of treatment length itself, thereby providing the most conservative estimate of the association.

Missing data, primarily from patient-reported outcome questionnaires that were not fully completed, were handled using a complete-case analysis. The varying sample sizes for each analysis, reflecting the number of complete cases, are reported in the respective results tables.

All statistical analyses were performed using the IBM SPSS software (version 29; IBM Corp., Armonk, NY, United States), and a correlation heat map was generated using R version 4.4.2 (R Foundation for Statistical Computing, Vienna, Austria).

## 3 Results

### 3.1 Patient characteristics

A total of 373 patients who were hospitalized at the Department of Acupuncture and Moxibustion at Kyung Hee University Korean Medicine Hospital for neck or low back pain under automobile insurance coverage between 1 September 2018 and 31 March 2023 were identified through the hospital’s Medical Records Management Team. Relevant diagnoses were coded using the International Classification of Diseases, Tenth Revision codes S13.4–S13.6 (sprain and strain of the cervical spine and associated structures) and S33.5–S33.7 (sprain and strain of the lumbar spine and related structures). Patient data were extracted from the hospital EMR.

**Figure 1:**
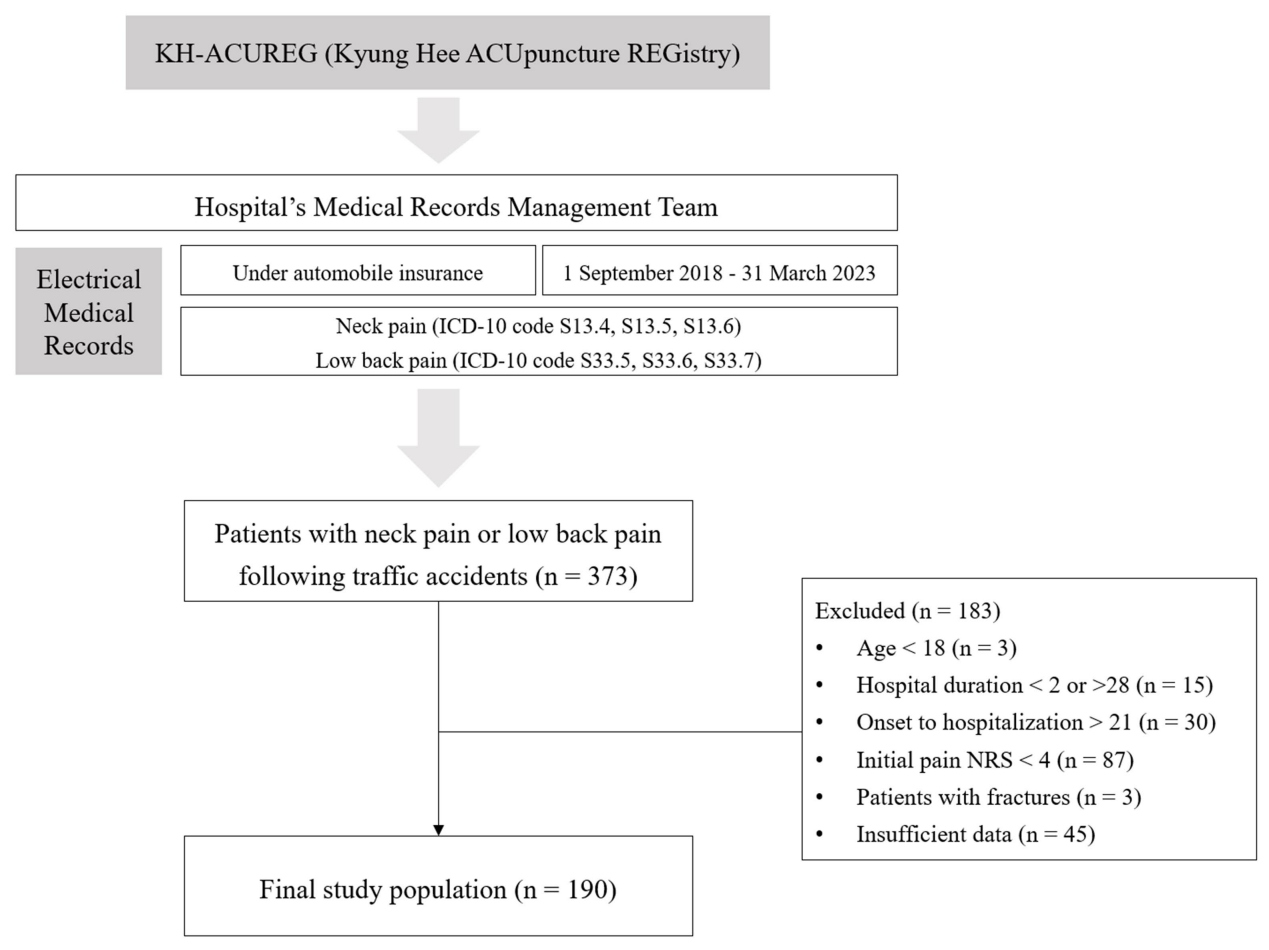
Flow chart of patient selection. *n*, number of patients; NRS, numeric rating scale; ICD-10, international classification of diseases, 10th revision; S13.4, sprain and strain of the cervical spine; S13.5, sprain and strain of the thyroid region; S13.6, sprain and strain of joints and ligaments of other and unspecified parts of the neck; S33.5, sprain and strain of the lumbar spine; S33.6, sprain and strain of the sacroiliac joint; S33.7, sprain and strain of other and unspecified parts of the lumbar spine and pelvis.

After applying the inclusion criteria, 183 patients were excluded. Consequently, 190 patients (118 women and 72 men) were included in the final analysis (Figure 1). Among them, 158 (97 women and 61 men) presented with neck pain, whereas 161 (100 women and 61 men) reported low back pain. A total of 129 patients (67.9% of the total study population) reported both neck and low back pain (Table 1).

**Table 1:** Demographic characteristics.

Variable		ALL (*n* = 190)	Neck pain (*n* = 158)	Low back pain (*n* = 161)
Sex	Male	72 (37.89%)	61 (38.61%)	61 (37.89%)
	Female	118 (62.11%)	97 (61.39%)	100 (62.11%)
Age (years)		47.08 ± 15.86	46.2 ± 14.91	46.42 ± 16.23
Height (cm)		164.08 ± 8.86	164.47 ± 8.83	164.08 ± 9.09
Weight (kg)		64.55 ± 13.29	65.06 ± 13.29	64.31 ± 13.65
BMI (kg/m^2^)		23.83 ± 3.53	23.91 ± 3.49	23.73 ± 3.63
Medical history	DM	16 (8.42%)	12 (7.59%)	13 (8.07%)
	HTN	35 (18.42%)	31 (19.62%)	29 (18.01%)
	Dyslipidemia	24 (12.63%)	20 (12.66%)	19 (11.8%)
	Cancer	10 (5.26%)	7 (4.43%)	8 (4.97%)
	Others	54 (28.42%)	45 (28.48%)	45 (27.95%)
Onset to hospitalization (days)		4.51 ± 4.47	3.91 ± 3.73	4.61 ± 4.52
Hospital day (days)		9.25 ± 4.49	9.13 ± 4.07	9.34 ± 4.55
Drug use		87 (45.79%)	69 (43.67%)	73 (45.34%)
Accident type	Rear end	98 (51.58%)	89 (56.33%)	80 (49.69%)
	Side impact	33 (17.37%)	27 (17.09%)	30 (18.63%)
	Frontal	23 (12.11%)	17 (10.76%)	17 (10.56%)
	Pedestrian	17 (8.95%)	10 (6.33%)	16 (9.94%)
	Motorcycle	7 (3.68%)	6 (3.8%)	5 (3.11%)
	Bicycle	3 (1.58%)	2 (1.27%)	3 (1.86%)
	Other	14 (7.37%)	12 (7.59%)	14 (8.7%)

DM, diabetes mellitus; HTN, hypertension. Data are reported as mean ± SD, unless otherwise indicated. Percentages were calculated based on the total number of patients in each subgroup. Overlapping counts were allowed for medical history and accident type. “Drug use” refers to the number of patients who were taking analgesics at the time of admission due to pain.

### 3.2 Changes in clinical outcome values

At the time of discharge, significant improvements were observed in several clinical measures compared to those at admission. Pain intensity, as measured using the NRS, decreased across all categories (all *p* < 0.001). The NDI improved significantly (*p* < 0.001). Although the ODI showed improvement, this change did not reach statistical significance (*p* = 0.166). Regarding quality of life, EQ-5D index scores improved significantly (*p* = 0.001). In terms of psychosomatic variables, PHQ-9 scores improved significantly (*p* = 0.038); however, no statistically significant changes were observed in ISI (*p* = 0.857), BAI (*p* = 0.085), or FSS (*p* = 0.065) (Table 2).

**Table 2:** Changes in clinical outcome values.

Variable	Admission	Discharge	Change	*p* value
Axial pain NRS (*n* = 190)	5.32 ± 1.39	3.23 ± 1.77	2.09 ± 1.84*	<0.001
Neck pain NRS (*n* = 158)	4.88 ± 1.36	2.67 ± 1.7	2.21 ± 1.82*	<0.001
Low back pain NRS (*n* = 161)	4.92 ± 1.61	2.98 ± 1.77	1.94 ± 1.78*	<0.001
NDI (*n* = 71)	19.56 ± 9.65	16.38 ± 8.13	3.18 ± 7.12*	<0.001
ODI (*n* = 63)	32.15 ± 18.51	29.43 ± 16.09	2.71 ± 15.36	0.166
ISI (*n* = 74)	11.66 ± 6.91	11.55 ± 6.18	0.11 ± 5.16	0.857
PHQ-9 (*n* = 78)	8.36 ± 6.33	7.28 ± 5.84	1.08 ± 4.51*	0.038
BAI (*n* = 75)	13.72 ± 13.28	12.27 ± 12.59	1.45 ± 7.2	0.085
FSS (*n* = 75)	4.31 ± 5.99	3.86 ± 4.35	0.46 ± 2.11	0.065
EQ-5D (*n* = 76)	8.47 ± 1.96	7.64 ± 2.38	0.83 ± 2.16*	0.001

An asterisk (*) indicates statistical significance (*p* < 0.05). Data are reported as mean ± SD. Change was calculated by subtracting the discharge value from the admission value. *n*, number of patients; NRS, numeric rating scale; NDI, neck disability index; ODI, Oswestry disability index; ISI, insomnia severity index; BAI, beck anxiety inventory; PHQ-9, patient health questionnaire-9; FSS, fatigue severity scale; EQ-5D, EuroQol five-dimensional questionnaire three-level version.

### 3.3 Correlations between baseline pain intensity and clinical measures at admission

**Figure 2:**
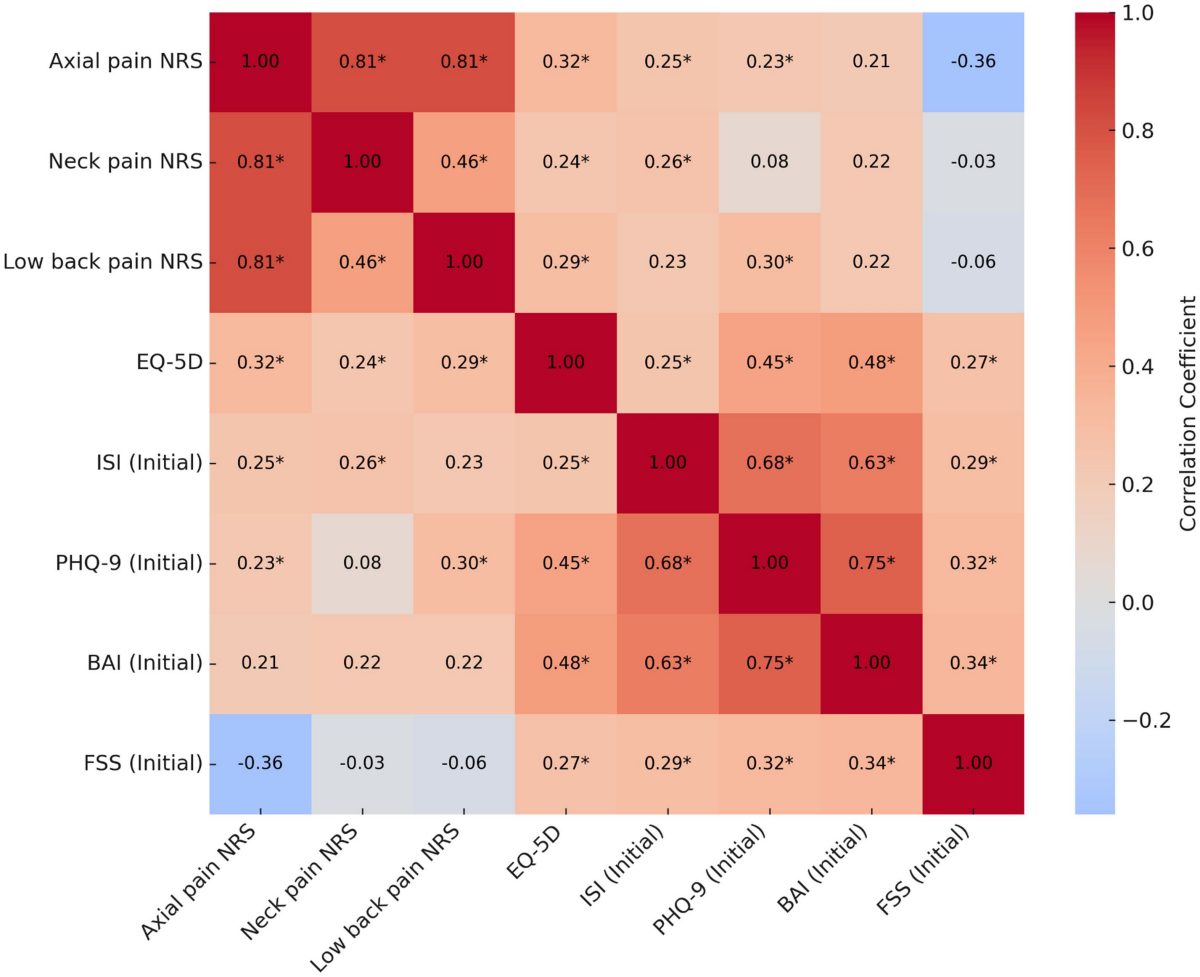
Correlation analysis between baseline pain intensity and clinical measures at admission. Asterisks (^*^) indicate statistical significance (*p* < 0.05). NRS, numeric rating scale; EQ-5D, EuroQol five-dimensional questionnaire, three-level version; ISI, insomnia severity index; PHQ-9, patient health questionnaire-9; BAI, beck anxiety inventory; FSS, fatigue severity scale.

The axial pain NRS at admission showed modest positive correlations with the EQ-5D index (*p* < 0.01), ISI (*p* < 0.05), and PHQ-9 (*p* < 0.05). Similarly, neck pain NRS scores showed significant positive correlations with the ISI (*p* < 0.05) and EQ-5D (*p* < 0.05), while low back pain NRS scores were positively associated with the PHQ-9 (*p* < 0.01). Among the psychosomatic variables, strong correlations were observed between the PHQ-9, BAI, and ISI scores. In addition, the FSS demonstrated weak to moderate positive correlations with other psychosomatic measures, including the ISI, PHQ-9, and BAI (Figure 2).

### 3.4 Univariable linear regression analyses of factors associated with baseline pain intensity at admission

Univariable linear regression analyses were conducted to identify the factors associated with baseline pain severity at admission. For axial pain, higher ISI scores (*β* = 0.05, 95% CI: 0.01–0.10, *p* = 0.0186), PHQ-9 scores (*β* = 0.05, 95% CI: 0.01–0.10, *p* = 0.0253), and age (*β* = 0.02, 95% CI: 0.00–0.03, *p* = 0.008) were positively associated with greater pain severity. In addition, height was inversely associated with axial pain scores (*β* = −0.03, 95% CI: −0.05 to −0.01, *p* = 0.0052), while a longer duration from accident onset to hospitalization was positively associated (*β* = 0.05, 95% CI: 0.01–0.10, *p* = 0.0152). Regarding neck pain, ISI (*β* = 0.06, 95% CI: 0.01–0.10, *p* = 0.0215) and BMI (*β* = 0.07, 95% CI: 0.01–0.13, *p* = 0.0196) were positively associated with NRS scores. For low back pain, PHQ-9 scores (*β* = 0.09, 95% CI: 0.02–0.15, *p* = 0.0096) and age (*β* = 0.02, 95% CI: 0.01–0.04, *p* = 0.002) showed positive significant associations, while height demonstrated a significant inverse association with pain severity (*β* = −0.04, 95% CI: −0.07 to −0.01, *p* = 0.0062). Other variables, including sex, weight, history of prior disease, use of pain medication, number of symptoms, and FSS, were not significantly associated with pain scores in any domain (Supplementary Table S2).

### 3.5 Univariable linear regression analyses of factors associated with post-treatment pain intensity at discharge

Several clinical and psychosomatic variables were significantly associated with the pain intensity at discharge. For axial pain NRS at discharge, greater pain reduction was significantly associated with longer hospitalization duration (*β* = −0.08, 95% CI: −0.14 to −0.02, *p* = 0.0079), greater decreases in BAI scores (*β* = −0.08, 95% CI: −0.13 to −0.03, *p* = 0.0037), reductions in PHQ-9 scores (*β* = −0.13, 95% CI: −0.21 to −0.05, *p* = 0.0012), and reductions in FSS scores (*β* = −0.20, 95% CI: −0.37 to −0.02, *p* = 0.0297). For neck pain NRS, only the reduction in PHQ-9 score was significantly associated with pain relief at discharge (*β* = −0.10, 95% CI: −0.18 to −0.02, *p* = 0.0196). Regarding low back pain NRS at discharge, higher ISI scores at admission were significantly associated with greater pain intensity (*β* = 0.07, 95% CI: 0.00–0.14, *p* = 0.047), whereas longer hospitalization duration (*β* = −0.08, 95% CI: −0.15 to −0.01, *p* = 0.022), greater reductions in BAI (*β* = −0.11, 95% CI: −0.16 to −0.06, *p* = 0.0001), PHQ-9 (*β* = −0.12, 95% CI: −0.21 to −0.02, *p* = 0.0186), and FSS scores (*β* = −0.21, 95% CI: −0.41 to −0.01, *p* = 0.037) were associated with greater pain improvement (Supplementary Table S3).

### 3.6 Multiple linear regression analyses of the association between psychosomatic variables and post-treatment pain intensity at discharge

Multiple linear regression analyses were performed using the three hierarchical models with increasing levels of adjustment. Model 1 was adjusted for baseline NRS scores, Model 2 included additional adjustments for demographic characteristics, and Model 3 included hospitalization duration.

Several psychosomatic variables were significantly associated with pain intensity at discharge. As additional covariates were progressively introduced across the models, the explanatory power (*R*^2^) increased, particularly in the axial and low back pain models. For axial pain NRS in Model 3, greater reductions were significantly associated with decreases in BAI (*p* = 0.0008), PHQ-9 (*p* = 0.0007), and FSS scores (*p* = 0.0179). ISI change reached statistical significance in Model 3 (*p* = 0.0245). For neck pain, NRS in Model 3 showed a significant association was observed only for the change in PHQ-9 (*p* = 0.0449). The other variables did not show consistent significance across the models. For low back pain NRS in Model 3, greater reductions in BAI (*p* = 0.0002), ISI (*p* = 0.0442), and FSS scores (*p* = 0.0451) were significantly associated with lower pain scores at discharge after adjusting for clinical and demographic covariates (Table 3).

**Table 3:** Multiple linear regression analyses of the association between psychosomatic variables and pain intensity at discharge.

		Axial pain NRS at discharge				Neck pain NRS at discharge				Low back pain NRS at discharge			
Model	Variable	*β* (95% CI)	*p* value	*R* ^2^	Δ*R*^2^	*β* (95% CI)	*p* value	*R* ^2^	Δ*R*^2^	*β* (95% CI)	*p* value	*R* ^2^	Δ*R*^2^
Model 1	ISI (Initial)	0.02 (−0.03, 0.07)	0.47	0.09		−0.01 (−0.07, 0.04)	0.58	0.07		0.05 (−0.02, 0.11)	0.15	0.21	
	BAI (Initial)	−0.01 (−0.04, 0.01)	0.34	0.10		−0.01 (−0.04, 0.01)	0.38	0.08		−0.02 (−0.05, 0.02)	0.33	0.21	
	PHQ-9 (Initial)	−0.03 (−0.09, 0.02)	0.22	0.16		−0.05 (−0.1, 0.0)	0.07	0.11		−0.02 (−0.09, 0.05)	0.49	0.21	
	FSS (Initial)	−0.02 (−0.08, 0.04)	0.58	0.09		−0.02 (−0.07, 0.04)	0.53	0.08		−0.02 (−0.09, 0.04)	0.50	0.21	
	ISI change	−0.04 (−0.12, 0.03)	0.24	0.07		−0.03 (−0.1, 0.04)	0.44	0.05		−0.04 (−0.12, 0.04)	0.34	0.16	
	BAI change	−0.08 (−0.12, −0.03)^*^	0.00	0.15		−0.02 (−0.07, 0.03)	0.38	0.08		−0.1 (−0.15, −0.05)^*^	0.00	0.33	
	PHQ-9 change	−0.12 (−0.2, −0.05)^*^	0.00	0.17		−0.09 (−0.17, −0.0)^*^	0.04	0.11		−0.09 (−0.19, −0.0)^*^	0.05	0.22	
	FSS change	−0.19 (−0.36, −0.01)^*^	0.04	0.10		−0.11 (−0.27, 0.05)	0.19	0.09		−0.15 (−0.35, 0.04)	0.12	0.19	
Model 2	ISI (Initial)	−0.0 (−0.06, 0.06)	0.95	0.17	0.08	−0.03 (−0.09, 0.03)	0.35	0.14	0.07	0.04 (−0.04, 0.11)	0.33	0.28	0.06
	BAI (Initial)	−0.02 (−0.05, 0.01)	0.20	0.20	0.10	−0.02 (−0.05, 0.01)	0.28	0.16	0.07	−0.02 (−0.05, 0.02)	0.35	0.29	0.08
	PHQ-9 (Initial)	−0.05 (−0.11, 0.01)	0.12	0.21	0.10	−0.06 (−0.11, 0.0)	0.05	0.19	0.08	−0.02 (−0.1, 0.06)	0.55	0.28	0.08
	FSS (Initial)	−0.02 (−0.09, 0.04)	0.48	0.19	0.09	−0.02 (−0.08, 0.04)	0.53	0.15	0.07	−0.03 (−0.1, 0.04)	0.38	0.29	0.08
	ISI change	−0.08 (−0.16, −0.0)^*^	0.04	0.20	0.14	−0.04 (−0.12, 0.04)	0.31	0.14	0.08	−0.09 (−0.18, 0.01)	0.07	0.29	0.13
	BAI change	−0.1 (−0.15, −0.04)^*^	0.00	0.30	0.15	−0.02 (−0.08, 0.03)	0.40	0.16	0.08	−0.12 (−0.17, −0.06)^*^	0.00	0.47	0.14
	PHQ-9 change	−0.15 (−0.22, −0.07)^*^	0.00	0.31	0.14	−0.1 (−0.18, −0.01)^*^	0.03	0.21	0.10	−0.11 (−0.21, −0.0)^*^	0.04	0.31	0.10
	FSS change	−0.25 (−0.43, −0.06)^*^	0.01	0.24	0.14	−0.12 (−0.3, 0.07)	0.21	0.18	0.08	−0.23 (−0.43, −0.02)^*^	0.03	0.34	0.15
Model 3	ISI (Initial)	−0.01 (−0.07, 0.05)	0.81	0.22	0.05	−0.03 (−0.09, 0.03)	0.38	0.15	0.01	0.04 (−0.04, 0.11)	0.31	0.30	0.03
	BAI (Initial)	−0.02 (−0.04, 0.01)	0.26	0.25	0.05	−0.01 (−0.04, 0.02)	0.37	0.17	0.01	−0.01 (−0.05, 0.03)	0.54	0.32	0.03
	PHQ-9 (Initial)	−0.04 (−0.1, 0.02)	0.19	0.25	0.04	−0.05 (−0.11, 0.01)	0.09	0.20	0.00	−0.01 (−0.09, 0.07)	0.78	0.31	0.03
	FSS (Initial)	−0.02 (−0.08, 0.05)	0.63	0.24	0.05	−0.02 (−0.08, 0.05)	0.62	0.17	0.019	−0.03 (−0.1, 0.05)	0.47	0.32	0.03
	ISI change	−0.09 (−0.17, −0.01)^*^	0.02	0.26	0.06	−0.04 (−0.12, 0.04)	0.31	0.15	0.01	−0.1 (−0.19, −0.0)^*^	0.04	0.33	0.04
	BAI change	−0.09 (−0.14, −0.04)^*^	0.00	0.34	0.04	−0.02 (−0.08, 0.04)	0.46	0.17	0.01	−0.11 (−0.16, −0.06)^*^	0.00	0.49	0.02
	PHQ-9 change	−0.14 (−0.21, −0.06)^*^	0.00	0.35	0.04	−0.09 (−0.18, −0.0)^*^	0.04	0.22	0.00	−0.1 (−0.2, 0.0)	0.05	0.35	0.03
	FSS change	−0.22 (−0.41, −0.04)^*^	0.02	0.28	0.04	−0.1 (−0.29, 0.08)	0.27	0.19	0.01	−0.21 (−0.41, −0.0)^*^	0.05	0.37	0.04

Model 1: adjusted for initial pain NRS.

Model 2: Adjusted for initial pain NRS score, sex, age, height, weight, BMI, medical history, drug use, number of chief complaints, and onset of hospitalization.

Model 3: Adjusted for initial pain NRS score, sex, age, height, weight, BMI, medical history, drug use, number of chief complaints, time from onset to hospitalization, and hospital stay.

Asterisks (*) indicate statistical significance (*p* < 0.05). NRS, numeric rating scale; ISI, insomnia severity index; PHQ-9, patient health questionnaire-9; BAI, beck anxiety inventory; FSS, fatigue severity scale.

### 3.7 Safety

Blood chemistry tests were conducted at both admission and discharge to assess the safety of TKM treatments. The mean values of the liver and kidney function markers, including AST, ALT, ALP, total bilirubin, BUN, and creatinine, remained within the normal reference ranges both before and after treatment. On average, AST and ALT levels showed stable values between admission and discharge (AST: 26.21 ± 12.93 to 23.72 ± 12.39 U/L; ALT: 23.41 ± 14.56 to 23.54 ± 13.89 U/L), and ALP increased slightly but remained well within the normal range (from 65.67 ± 20.54 to 71.65 ± 29.74 U/L). Renal function markers such as BUN and creatinine remained stable with no significant changes. Less than 10% of patients showed mild elevations in liver and renal function tests at discharge. However, these values remained close to the normal range and were not associated with any clinical symptoms or adverse outcomes (Supplementary Table S4). None of these deviations was considered clinically significant, and no additional medical interventions were required. Furthermore, a review of the medical records revealed no documented cases of mild adverse events potentially attributable to TKM treatment, such as petechiae, swelling, or erythema. In addition, no recorded instances of serious adverse events were noted, including anaphylactic shock, during hospitalization.

## 4 Discussion

Our retrospective cohort study demonstrates that an integrative inpatient approach, prioritizing Traditional Korean Medicine (TKM), is a clinically effective and safe strategy for managing acute neck and low back pain after traffic accidents. Patients experienced significant reductions in pain alongside improvements in physical function and overall quality of life. Importantly, our findings reveal a strong, independent association between physical recovery and the resolution of psychosomatic symptoms. Improvements in anxiety, depression, fatigue, and insomnia were linked to greater pain relief, suggesting that concurrent management of psychological distress may be essential for optimizing recovery outcomes. This underscores the value of a holistic, integrative model in the acute post-accident phase to effectively manage pain and potentially mitigate the transition to chronic conditions. This multi-dimensional assessment aligns with contemporary rehabilitation paradigms, such as the biopsychosocial model, and allows for a more comprehensive, patient-centered interpretation of the clinical outcomes (37).

The most clinically significant finding of this study was the substantial reduction in pain intensity and the concurrent improvement in neck-related function. Specifically, the mean reductions in the axial pain NRS score (2.09 points) and neck pain NRS score (2.21 points) were substantial. These reductions exceed the commonly accepted threshold for a Minimally Clinically Important Difference (MCID)—typically a change of 2 points or 30%—which signifies a level of pain relief that is perceptible and meaningful to patients (38). In contrast, the improvements in psychosomatic measures were less consistent. Although depression scores (PHQ-9) and quality of life (EQ-5D) improved significantly, changes in insomnia (ISI), anxiety (BAI), and fatigue (FSS) did not reach statistical significance, despite trending positively.

The non-significant changes in the ISI, BAI, and FSS warrant a nuanced interpretation. While TKM interventions such as acupuncture are known to be effective for these psychosomatic symptoms (39–41), their therapeutic effects may be less pronounced in the context of acute traumatic pain. It is plausible that during the brief hospitalization period (mean 9.25 days), the primary therapeutic focus for both patients and clinicians was the management of severe acute pain. This immediate physical crisis likely overshadowed the underlying psychological distress, which might have become a more central therapeutic target in a chronic pain context. This highlights the different temporal dynamics of recovery; acute nociceptive pain can be rapidly modulated, whereas resolving more complex psychological and behavioral patterns requires a longer therapeutic duration (37). Achieving significant changes in these domains often benefits from continued care that may include targeted psychological interventions, such as cognitive-behavioral therapy, alongside somatically-focused treatments (42). Therefore, the lack of significant findings likely reflects the study’s acute-phase, short-term design rather than a limitation of the TKM interventions themselves.

At baseline, pain severity after TA showed a modest but significant association with psychosomatic symptoms. Correlation analysis revealed that pain intensity was positively associated with insomnia (indicated by ISI) and depressive symptoms (indicated by PHQ-9). Neck pain was correlated with ISI scores, whereas low back pain was significantly associated with PHQ-9 scores. These findings suggest that psychological distress, particularly sleep disturbance and depression, is associated with the initial perception of musculoskeletal pain, with variations depending on the region of pain. Moreover, poor sleep quality and depression interact bidirectional with pain, forming reinforcing cycles in which each factor exacerbates the others, ultimately increasing pain perception (43). Neuroimaging findings support the notion that depression and pain interact at a neural level. A recent activation-likelihood estimation meta-analysis demonstrated that pain accompanied by depression is associated with the activation of the right amygdala, whereas depression accompanied by pain is linked to the activation of the left dorsolateral prefrontal cortex (44). Univariable linear regression analyses also supported these associations and identified several demographic factors associated with pain severity. Higher age was associated with greater pain severity, which may reflect the increased injury susceptibility of older adults compared with younger individuals, even in low-impact collisions (45). In contrast, greater height was inversely associated with pain intensity, particularly in patients with low back pain. However, other factors such as sex, body weight, BMI, and medical history were not significantly associated with the baseline pain scores. Although previous studies have proposed that sex (46), BMI (47), and medical history before an accident may affect post-accident pain outcomes (48), these associations have not been established in the existing literature.

Furthermore, neither the use of pain medication at admission nor the number of reported painful body regions showed a significant association with the NRS scores. This suggests that patients’ subjective pain intensity may be shaped more by individual psychosomatic responses than by the extent of physical discomfort or pharmacological intervention at the time of the hospital visit. Although previous studies have suggested that a greater number of painful sites may be associated with higher pain intensity (49, 50), this relationship was not observed in the current study. The number of symptomatic regions may be less influential in cases of acute pain with a clear onset. Instead, situational and psychological factors present at the time of injury may play a dominant role in shaping the initial pain perception.

Regression analyses revealed several notable patterns regarding factors associated with pain intensity at discharge. In the univariable linear regression analyses, higher baseline ISI scores were significantly associated with higher discharge NRS scores, suggesting poorer pain improvement. However, other baseline psychosomatic variables, including the PHQ-9, BAI, and FSS, showed no significant associations, implying that the initial psychosomatic status may have limited predictive value for short-term recovery from acute traumatic pain. Conversely, longer hospitalization duration was associated with greater pain reduction, highlighting the importance of adequate treatment time and intensity in managing post-accident musculoskeletal pain. Greater improvements in the BAI, PHQ-9, and FSS scores during hospitalization were significantly associated with lower NRS scores at discharge, particularly in patients with low back pain. In multiple linear regression analyses using progressively adjusted models, changes in the PHQ-9, BAI, and FSS scores consistently demonstrated significant associations with pain relief at discharge. These associations remained robust even after adjusting for demographic characteristics, clinical variables, and hospitalization duration. The ISI change was not significantly associated with pain outcomes in the univariable analyses or the initial multivariate model, which was adjusted only for baseline pain intensity. However, when demographic and clinical factors as well as hospital stay duration were considered, the association between ISI change and pain improvement was statistically significant. This suggests that improvements in sleep disturbance may significantly contribute to pain recovery when considered in a broader clinical context. Furthermore, as additional covariates were included across the models, the *R*^2^ values increased, indicating that psychosomatic recovery during hospitalization contributed significantly to pain outcomes. Altogether, these findings suggest that addressing psychological symptoms such as sleep disturbance, mood, and fatigue, along with physical treatment, may enhance the overall recovery of patients experiencing TA-related pain.

In addition to demonstrating clinical improvements, this study provides preliminary support for the safety of TKM treatment in patients hospitalized for TA-related musculoskeletal pain. Laboratory findings, including liver and kidney function parameters, remained within the normal ranges before and after treatment in most cases. Although a small number of patients had values slightly exceeding the reference limits at discharge, none were considered clinically significant or required medical intervention. Furthermore, no serious adverse events occurred throughout the treatment period, and no cases of minor events attributable to TKM treatments were documented in the medical records. These findings suggest that TKM interventions may be safe and well tolerated in inpatient settings. These findings emphasize the importance of addressing psychological symptoms such as depression, anxiety, fatigue, and sleep disturbances, alongside physical pain, in patients experiencing acute musculoskeletal injuries following TAs. Given the complex interplay between physical and psychosomatic factors, a treatment approach that simultaneously targets both domains is essential for optimal recovery. TKM, with its emphasis on holistic and individualized care, may be particularly well suited for managing such multifaceted conditions. Recent studies have increasingly provided evidence supporting the therapeutic efficacy of TKM in managing psychological factors such as anxiety (51, 52), depression (53), and post-traumatic stress disorder (54). Furthermore, the observed association between longer hospitalization and greater pain reduction suggests that sufficient access to TKM treatment may be essential for achieving clinically meaningful improvements in this population.

This study had several strengths. This study investigated patient outcomes after TKM treatment in the acute phase of TA-related musculoskeletal pain using real-world clinical data. Although the retrospective design limited the ability to establish a causal relationship between treatment and outcomes, our findings provide valuable insights into patient responses during hospitalization. This study examined various psychosomatic and clinical variables that may influence pain outcomes. These results may inform clinical decision-making by encouraging practitioners to consider psychosomatic symptoms such as depression, anxiety, and fatigue when managing acute TA-related pain in patients. Additionally, treatment safety was evaluated through laboratory monitoring and clinical observation.

However, this study had certain limitations. As a retrospective analysis, it is inherently prone to bias due to the absence of a control group, potential confounding factors, and limitations of data completeness and consistency. Moreover, the data were collected over several years from routine clinical records. Consequently, the quality and details of the documentation may vary depending on the practitioner. Minor or transient adverse events may have been underreported or inconsistently recorded, which could potentially limit the assessment of treatment safety. Furthermore, missing data, primarily from patient-reported outcome questionnaires, were handled using a complete-case analysis. This approach may have reduced statistical power and introduced bias if the data were not missing completely at random; however, it was chosen for its straightforwardness and transparency.

## 5 Conclusion

In this retrospective cohort study, the clinical outcomes and safety of TKM as part of integrative care were evaluated in patients hospitalized for acute neck pain and low back pain following TAs. Pain reduction was observed without serious adverse events, indicating that the treatment may be both effective and safe in clinical settings. Additionally, improvements in psychosomatic variables such as depression, anxiety, and fatigue were observed to be significantly associated with greater pain relief. These findings suggest that consideration of the psychosomatic status and physical symptoms is essential for the management of post-accident musculoskeletal pain.

## Data Availability

The raw data supporting the conclusions of this article will be made available by the authors without undue reservation.
