# Supplementary material for: Clinical outcomes and psychosomatic correlates of integrative inpatient care based on Traditional Korean Medicine for acute neck and low back pain following traffic accidents: a retrospective cohort study
Source: Front Med (Lausanne). 2025 Nov 13;12:1684034. doi: 10.3389/fmed.2025.1684034 (PMC12657461; doi:10.3389/fmed.2025.1684034)
Supplement: Supplementary file 1 [file Table_1.docx]

Supplementary Material

# Supplementary Tables

## Table S1. Herbal medicine and composition

| Decoction | *Tonghyeol hwallak-tang*  (統血活絡湯) |  | *Tonghyeolhoesu-san*  (統血回首散) |  | *Tonghyeol ssanghwa-tang*  (統血雙和湯) |  |
| --- | --- | --- | --- | --- | --- | --- |
| Composition* | *Chelidonii Herba*  (白屈菜) | 8 g | *Citri Unshius Pericarpium*  (陳皮) | 6 g | *Paeoniae Radix*  (芍藥) | 10 g |
|  | *Corydalis Tuber*  (玄胡索) | 8 g | *Ephedrae Herba*  (麻黃) | 6 g | *Zizyphi Fructus*  (大棗) | 6 g |
|  | *Chaenomelis Fructus*  (木瓜) | 8 g | *Linderae Radix*  (烏藥) | 6 g | *Zingiberis Rhizoma Recens*  (生薑) | 6 g |
|  | *Zizyphi Fructus*  (大棗) | 6 g | *Batryticatus Bombyx*  (白殭蠶) | 4 g | *Cnidii Rhizoma*  (川芎) | 4 g |
|  | *Zingiberis Rhizoma Recens*  (生薑) | 6 g | *Cnidii Rhizoma*  (川芎) | 4 g | *Angelicae Gigantis Radix*  (當歸) | 4 g |
|  | *Clematidis Radix*  (威靈仙) | 6 g | *Araliae Continentalis Radix*  (獨活) | 4 g | *Astragali Radix*  (黃芪) | 4 g |
|  | *Carthami Flos*  (紅花) | 5 g | *Platycodonis Radix*  (桔梗) | 4 g | *Rehmanniae Radix Preparata*  (熟地黃) | 4 g |
|  | *Atractylodis Rhizoma*  (蒼朮) | 3 g | *Osterici seu Notopterygii Radix et Rhizoma* (羌活) | 4 g | *Glycyrrhizae Radix et Rhizoma*  (甘草) | 3 g |
|  | *Angelicae Gigantis Radix*  (當歸) | 3 g | *Aurantii Fructus Immaturus*  (枳實) | 4 g | *Cinnamomi Cortex*  (肉桂) | 3 g |
|  | *Araliae Continentalis Radix* (獨活) | 3 g | *Chaenomelis Fructus*  (木瓜) | 4 g | *Persicae Semen*  (桃仁) | 2 g |
|  | *Osterici seu Notopterygii Radix et Rhizoma* (羌活) | 3 g | *Persicae Semen*  (桃仁) | 2 g | *Carthami Flos*  (紅花) | 2 g |
|  | *Rehmanniaeth Radix*  (地黃) | 3 g | *Glycyrrhizae Radix et Rhizoma*  (甘草) | 2 g |  |  |
|  | *Paeoniae Radix*  (芍藥) | 3 g | *Zingiberis Rhizoma*  (乾薑) | 2 g |  |  |
|  | *Citri Unshius Pericarpium*  (陳皮) | 3 g | *Carthami Flos*  (紅花) | 2 g |  |  |
|  | *Myrrha*  (沒藥) | 3 g |  |  |  |  |
|  | *Olibanum*  (乳香) | 3 g |  |  |  |  |
|  | *Persicae Semen*  (桃仁), | 2 g |  |  |  |  |
|  | *Glycyrrhizae Radix et Rhizoma*  (甘草), | 2 g |  |  |  |  |
|  | *Amomi Fructus*  (砂仁) | 2 g |  |  |  |  |

* The composition was decocted in 300 mL of water to produce an extract, which was then divided into three 100 mL portions. The patient received one pack three times daily.

## Table S2. Univariable Linear Regression of Factors with Baseline Pain Intensity at Admission

|  | **Axial pain NRS (Baseline)** | | | **Neck pain NRS (Baseline)** | | | **Low back pain NRS (Baseline)** | | |
| --- | --- | --- | --- | --- | --- | --- | --- | --- | --- |
| **Variable** | **β (95% CI)** | **P value** | **R^2^** | **β (95% CI)** | **P value** | **R^2^** | **β (95% CI)** | **P value** | **R^2^** |
| ISI (Initial) | 0.05 (0.01, 0.1)* | 0.0186 | 0.0628 | 0.06 (0.01, 0.1)* | 0.0215 | 0.0668 | 0.06 (-0.0, 0.13) | 0.0506 | 0.0543 |
| PHQ-9  (Initial) | 0.05 (0.01, 0.1)* | 0.0253 | 0.0544 | 0.02 (-0.03, 0.07) | 0.477 | 0.0063 | 0.09 (0.02, 0.15)* | 0.0096 | 0.0885 |
| BAI (Initial) | 0.02 (-0.0, 0.05) | 0.0526 | 0.0415 | 0.02 (-0.0, 0.05) | 0.0514 | 0.0466 | 0.03 (-0.0, 0.06) | 0.0638 | 0.0469 |
| FSS (Initial) | -0.01 (-0.06, 0.05) | 0.7378 | 0.0013 | -0.01 (-0.06, 0.05) | 0.7928 | 0.0009 | -0.02 (-0.09, 0.05) | 0.591 | 0.004 |
| Sex | 0.26 (-0.15, 0.67) | 0.2075 | 0.0084 | 0.1 (-0.35, 0.54) | 0.6671 | 0.0012 | 0.34 (-0.18, 0.86) | 0.1982 | 0.0104 |
| Age | 0.02 (0.0, 0.03)* | 0.008 | 0.0368 | 0.01 (-0.01, 0.02) | 0.246 | 0.0087 | 0.02 (0.01, 0.04)* | 0.002 | 0.0587 |
| Height | -0.03 (-0.05, -0.01)* | 0.0052 | 0.0407 | -0.01 (-0.04, 0.01) | 0.2956 | 0.0071 | -0.04 (-0.07, -0.01)* | 0.0062 | 0.0463 |
| Weight | -0.01 (-0.02, 0.01) | 0.483 | 0.0026 | 0.01 (-0.01, 0.02) | 0.3465 | 0.0057 | -0.02 (-0.03, 0.0) | 0.1026 | 0.0167 |
| BMI | 0.04 (-0.02, 0.09) | 0.1794 | 0.0096 | 0.07 (0.01, 0.13)* | 0.0196 | 0.0346 | 0.0 (-0.07, 0.07) | 0.9998 | 0 |
| Medical history | 0.33 (-0.07, 0.73) | 0.1017 | 0.0142 | -0.05 (-0.48, 0.38) | 0.8224 | 0.0003 | 0.41 (-0.09, 0.91) | 0.1101 | 0.016 |
| Drug use | -0.01 (-0.41, 0.39) | 0.9606 | 0 | -0.12 (-0.55, 0.32) | 0.5995 | 0.0018 | 0.07 (-0.43, 0.58) | 0.7709 | 0.0005 |
| Onset to hospitalization | 0.05 (0.01, 0.1)* | 0.0152 | 0.031 | 0.05 (-0.01, 0.11) | 0.0801 | 0.0196 | 0.06 (-0.0, 0.11) | 0.0503 | 0.0239 |
| N of Symptoms | 0.10 (-0.06, 0.25) | 0.2123 | 0.0083 | 0.01 (-0.17, 0.19) | 0.8998 | 0.0001 | 0.00 (-0.20, 0.20) | 0.9858 | 0 |

An asterisk (*) indicates statistical significance (p < 0.05). NRS: Numeric Rating Scale; ISI: Insomnia Severity Index; PHQ-9: Patient Health Questionnaire–9; BAI: Beck Anxiety Inventory; FSS: Fatigue Severity Scale; BMI: Body Mass Index; N: Number. Drug use refers to taking analgesics at the time of admission due to pain.

## Table S3. Univariable Linear Regression Analyses of Factors Associated with Post-treatment Pain Intensity at Discharge

|  | **Axial pain NRS (Discharge)** | | | **Neck pain NRS (Discharge)** | | | **Low back pain NRS (Discharge)** | | |
| --- | --- | --- | --- | --- | --- | --- | --- | --- | --- |
| **Variable** | **β (95% CI)** | **P value** | **R^2^** | **β (95% CI)** | **P value** | **R^2^** | **β (95% CI)** | **P value** | **R^2^** |
| ISI (Initial) | 0.04 (-0.02, 0.09) | 0.187 | 0.0204 | 0.0 (-0.05, 0.05) | 0.9575 | 0 | 0.07 (0.0, 0.14)* | 0.047 | 0.0576 |
| PHQ-9 (Initial) | -0.02 (-0.07, 0.04) | 0.5883 | 0.0033 | -0.04 (-0.1, 0.01) | 0.1102 | 0.0316 | 0.02 (-0.06, 0.09) | 0.6831 | 0.0024 |
| BAI (Initial) | -0.0 (-0.03, 0.02) | 0.7241 | 0.0014 | -0.0 (-0.03, 0.02) | 0.7501 | 0.0013 | -0.0 (-0.04, 0.04) | 0.9508 | 0.0001 |
| FSS (Initial) | -0.02 (-0.08, 0.04) | 0.5163 | 0.0048 | -0.02 (-0.08, 0.04) | 0.4907 | 0.006 | -0.03 (-0.1, 0.04) | 0.3733 | 0.0113 |
| Sex | 0.1 (-0.43, 0.63) | 0.7121 | 0.0007 | 0.23 (-0.33, 0.78) | 0.4196 | 0.0043 | 0.06 (-0.53, 0.65) | 0.8309 | 0.0003 |
| Age | -0.0 (-0.02, 0.01) | 0.7595 | 0.0005 | 0.0 (-0.02, 0.02) | 0.9896 | 0 | 0.0 (-0.01, 0.02) | 0.7519 | 0.0007 |
| Height | -0.0 (-0.03, 0.02) | 0.7858 | 0.0004 | -0.0 (-0.03, 0.03) | 0.9242 | 0.0001 | -0.0 (-0.04, 0.03) | 0.8019 | 0.0004 |
| Weight | 0.01 (-0.01, 0.03) | 0.5593 | 0.0018 | -0.0 (-0.02, 0.02) | 0.8827 | 0.0001 | 0.01 (-0.01, 0.03) | 0.2675 | 0.0081 |
| BMI | 0.04 (-0.03, 0.11) | 0.259 | 0.0069 | 0.0 (-0.07, 0.08) | 0.9321 | 0 | 0.06 (-0.01, 0.14) | 0.1011 | 0.0177 |
| Medical History | 0.05 (-0.46, 0.57) | 0.836 | 0.0002 | -0.07 (-0.61, 0.47) | 0.8004 | 0.0004 | 0.25 (-0.32, 0.81) | 0.3948 | 0.0048 |
| N of Symptoms | 0.13 (-0.06, 0.33) | 0.1799 | 0.0097 | 0.04 (-0.18, 0.26) | 0.7338 | 0.0008 | 0.01 (-0.23, 0.24) | 0.9558 | 0 |
| Drug Use | 0.07 (-0.44, 0.58) | 0.7841 | 0.0004 | -0.1 (-0.64, 0.44) | 0.7159 | 0.0009 | 0.13 (-0.44, 0.7) | 0.6624 | 0.0013 |
| Onset to hospitalization | -0.0 (-0.06, 0.05) | 0.9294 | 0 | -0.04 (-0.12, 0.03) | 0.2425 | 0.0089 | 0.02 (-0.04, 0.08) | 0.5414 | 0.0025 |
| Hospital day | -0.08 (-0.14, -0.02)* | 0.0079 | 0.0375 | -0.06 (-0.13, 0.01) | 0.0759 | 0.0204 | -0.08 (-0.15, -0.01)* | 0.022 | 0.0343 |
| BAI change | -0.08 (-0.13, -0.03)* | 0.0037 | 0.1113 | -0.02 (-0.07, 0.03) | 0.389 | 0.0118 | -0.11 (-0.16, -0.06)* | 0.0001 | 0.2287 |
| ISI change | -0.04 (-0.12, 0.03) | 0.2553 | 0.0182 | -0.02 (-0.09, 0.05) | 0.5181 | 0.0068 | -0.06 (-0.15, 0.02) | 0.1474 | 0.0371 |
| PHQ-9 change | -0.13 (-0.21, -0.05)* | 0.0012 | 0.1315 | -0.1 (-0.18, -0.02)* | 0.0196 | 0.0798 | -0.12 (-0.21, -0.02)* | 0.0186 | 0.0889 |
| FSS change | -0.2 (-0.37, -0.02)* | 0.0297 | 0.0639 | -0.12 (-0.28, 0.04) | 0.1502 | 0.0326 | -0.21 (-0.41, -0.01)* | 0.037 | 0.0741 |

An asterisk (*) indicates statistical significance (p < 0.05). NRS: Numeric Rating Scale; ISI: Insomnia Severity Index; PHQ-9: Patient Health Questionnaire–9; BAI: Beck Anxiety Inventory; FSS: Fatigue Severity Scale; N: Number

## Table S4. Blood Chemistry Pre- (Admission) and Post-Treatment (Discharge) and Out-of-Range Incidence at Discharge

| Outcome | Admission (Mean ± SD) | Discharge (Mean ± SD) | Normal range | n out of normal range at discharge (%) |
| --- | --- | --- | --- | --- |
| AST (U/L) | 26.21 ± 12.93 | 23.72 ± 12.39 | 0–50 | 2/64 (3.1%) |
| ALT (U/L) | 23.41 ± 14.56 | 23.54 ± 13.89 | 0–50 | 6/63 (9.5%) |
| ALP (U/L) | 65.67 ± 20.54 | 71.65 ± 29.74 | 30–120 | 2/60 (3.3%) |
| Total Bilirubin (mg/dL) | 0.65 ± 0.34 | 0.6 ± 0.25 | 0.3–1.2 | 2/53 (3.8%) |
| BUN (mg/dL) | 15.19 ± 7.62 | 15.18 ± 10.54 | 8–20 | 6/62 (9.7%) |
| Creatinine (mg/dL) | 0.79 ± 0.52 | 0.85 ± 0.91 | 0.67–1.20 | 2/63 (3.2%) |

AST: Aspartate Aminotransferase; ALT: Alanine Aminotransferase; ALP: Alkaline Phosphatase; BUN: Blood Urea Nitrogen; n: number of patients. Percentages (%) are based on the number of patients with available results at discharge.
